# Supplementary material for: Increased Azithromycin Susceptibility of Multidrug-Resistant Gram-Negative Bacteria on RPMI-1640 Agar Assessed by Disk Diffusion Testing
Source: Antibiotics (Basel). 2020 Apr 29;9(5):218. doi: 10.3390/antibiotics9050218 (PMC7277395; doi:10.3390/antibiotics9050218)

Table S1: Concentration of antibiotic disks

Concentration of antibiotic disks used for disk diffusion assay.

IU: international units, disk size: 6 mm

	Antibiotic	Disk concentration
**Macrolides**	Azithromycin	15 µg
	Erythromycin	15 IU
	Clarithromycin	15 µg
	Tobramycin	10 µg
	Cefepime	30 µg
	Tetracycline	30 IU
	Tigecycline	15 µg

Table S2: Decrease in resistance towards azithromycin in RPMI-1640 or MH containing $NaHCO_3$.

Azithromycin MICs of four strains per species (*K. pneumoniae, E. coli, E. cloacae* and *P. aeruginosa*) were measured in RPMI-1640 and MH with and without $NaHCO_3$ [2 g/L]. The decrease in resistance towards azithromycin is shown as fold change reduction of the MIC in RPMI or MH plus $NaHCO_3$ as compared to MH without $NaHCO_3$.

species	Fold change reduction of azithromycin MIC in RPMI-1640 or MH + $NaHCO_3$ as compared to MH alone	
	RPMI	MH + $NaHCO_3$
K. pneumoniae	8	8
	64	32
	128	16
	1	1
E. coli	256	16
	64	16
	64	32
	256	32
E. cloacae	128	16
	32	16
	32	16
	128	16
P. aeruginosa	32	8
	1	1
	32	4
	1	1

Table S3: Median erythromycin and tetracycline inhibition zone diameters of *Enterobacteriaceae* and *P. aeruginosa* on MH and RPMI-1640 agar.

Erythromycin and tetracycline susceptibility of *Enterobacteriaceae* (28 *E. coli*, 24 *E. cloacae* and 26 *K. pneumonia*) and 55 *P. aeruginosa* isolates was measured by disk diffusion on MH and RPMI-1640 agar. Data show median + interquartile range, Nonparametric Mann-Whitney Test, ns: not significant** <0.001, *** <0.0001. IU: international units, disk size: 6 mm, 15 IU erythromycin, 30 IU tetracycline.

erythromycin

	**species**	**MH [mm]**	**RPMI [mm]**
Enterobacteriaceae	*E. coli*	6 (6-6)	7.5 (6-9.5)***
	E. cloacae	6 (6-6)	6 (6-6) ns
	K. pneumoniae	6 (6-6)	6 (6-7.1)**
	P. aeruginosa	6 (6-6)	6 (6-6.6) ns

tetracycline

	**species**	**MH [mm]**	**RPMI [mm]**
Enterobacteriaceae	*E. coli*	11.5 (6-27.5)	12.25 (6-27.4) ns
	E. cloacae	23 (19.1-24.5)	22.4 (18.6-23.7) ns
	K. pneumoniae	24.3 (20.6-25.6)	24.8 (18.4-26.1) ns
	P. aeruginosa	14 (7.2-19.6)	10.2 (6.2-15.7) ns

Figure S1: Increased azithromycin susceptibility in carbapenemase-positive *Enterobacteriaceae* on RPMI-1640 agar as compared to MH agar.

Azithromycin susceptibility of various carbapenemase-positive *Enterobacteriaceae* isolates (2 *E. coli*, 14 *K. pneumoniae*, 1 *Citrobacter koseri*, 1 *Proteus mirabilis,* 1 *Morganella morganii*) and of one carbapenemase-positive *P. aeruginosa* isolate was measured in MH (filled bars) and RPMI-1640 (open bars) media by disk diffusion. Each isolate was tested in two independent experiments. Data display mean (± SD for *K. pneumoniae*). Disk size: 6 mm, 15 µg azithromycin.

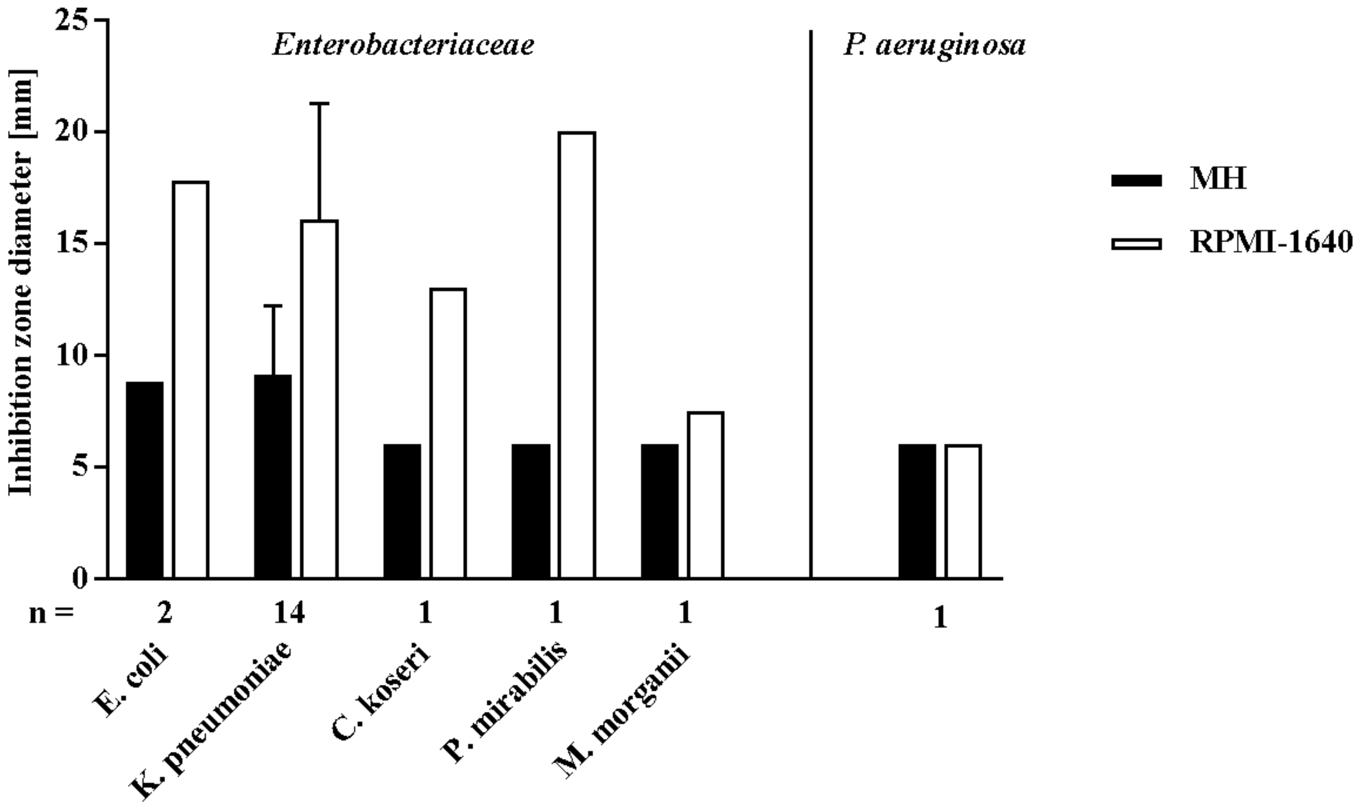

Supplement: Supplementary file 1 [file antibiotics-09-00218-s001.pdf]
